# Spaceborne Sparse SAR Imaging Mode Design: From Theory to Implementation

**DOI:** 10.3390/s25133888

**Published:** 2025-06-22

**Authors:** Yufan Song, Hui Bi, Fuxuan Cai, Guoxu Li, Jingjing Zhang, Wen Hong

**Affiliations:** 1 College of Electronic and Information Engineering, Nanjing University of Aeronautics and Astronautics, Nanjing 211106, China; 2 Key Laboratory of Radar Imaging and Microwave Photonics, Ministry of Education, Nanjing University of Aeronautics and Astronautics, Nanjing 211106, China; 3 Aerospace Information Research Institute, Chinese Academy of Sciences, Beijing 100094, China

**Keywords:** synthetic aperture radar (SAR), mode design, sparse SAR imaging, beam position

## Abstract

To satisfy the requirement of the modern spaceborne synthetic aperture radar (SAR) system, SAR imaging mode design makes a trade-off between resolution and swath coverage by controlling radar antenna sweeping. Existing spaceborne SAR systems can perform earth observation missions well in various modes, but they still face challenges in data acquisition, storage, and transmission, especially for high-resolution wide-swath imaging. In the past few years, sparse signal processing technology has been introduced into SAR to try to solve these problems. In addition, sparse SAR imaging shows huge potential to improve system performance, such as offering wider swath coverage and higher recovered image quality. In this paper, the design scheme of spaceborne sparse SAR imaging modes is systematically introduced. In the mode design, we first design the beam positions of the sparse mode based on the corresponding traditional mode. Then, the essential parameters are calculated for system performance analysis based on radar equations. Finally, a sparse SAR imaging method based on mixed-norm regularization is introduced to obtain a high-quality image of the considered scene from the data collected by the designed sparse modes. Compared with the traditional mode, the designed sparse mode only requires us to obtain a wider swath coverage by reducing the pulse repetition rate (PRF), without changing the existing on-board system hardware. At the same time, the reduction in PRF can significantly reduce the system data rate. The problem of the azimuth ambiguity signal ratio (AASR) increasing from antenna beam scanning can be effectively solved by using the mixed-norm regularization-based sparse SAR imaging method.

## 1. Introduction

Synthetic aperture radar (SAR) is an active microwave imaging technology with all-time and all-weather surveillance ability. Nowadays, it has been widely used in mapping, military warning, and disaster surveillance applications [1,2,3]. SAR systems are typically installed on either airborne or spaceborne platforms. Compared with airborne systems, spaceborne SAR has better performance, including wider coverage, better information capture, and improved battlefield survivability. In the development of spaceborne SAR, achieving high-resolution and wide-swath imaging is a long-standing challenge. However, because of the limitation of antenna size, high resolution and wide swath are a pair of contradictions [4]. In recent years, several works have been proposed to tackle this challenge, which can be categorized into two groups: multichannel technology and imaging mode design [5]. Multichannel technology focuses on minimizing the sampling requirements in the time domain such as multidimensional waveform encoding (MWE) [6] and displaced phase center antenna (DPCA) [7]. These methods are efficient but have high requirements for data transmission and power consumption. On the other hand, SAR imaging mode design addresses the compromise between resolution and swath width by controlling antenna beam pointing. By using different imaging modes, SAR systems can satisfy the requirements of different mapping tasks. This approach enables the optimization of imaging parameters to strike a balance between high-resolution and wide-swath coverage.

The most common SAR imaging modes are Stripmap [1,2], Spotlight [8,9], and Scan [10,11]. Spotlight mode achieves long-term observation of a specific area by sacrificing azimuth swath coverage, resulting in high-resolution imaging. In contrast, Scan utilizes burst mode to achieve wide-swath observation by switching the antenna beam among subswaths. Building on these fundamental ones, several other imaging modes have been developed, including Terrain Observation by Progressive Scans (TOPS) [12,13,14], Mosaic [15,16,17], and Sliding Spotlight [18,19,20]. As a novel burst imaging mode, TOPS tackles the challenge of wide-swath imaging [12]. Unlike Scan, it achieves the same swath coverage while reducing scalloping by illuminating all targets with the antenna azimuth pattern (AAP) [13,14]. Sliding Spotlight, developed from Spotlight, is a high-resolution imaging mode that provides longer target irradiation time for capturing higher-resolution images [18,19,20]. Additionally, it offers wider azimuth swath coverage than Spotlight. Mosaic mode is a novel imaging mode designed to achieve both high-resolution and wide-swath observation [15]. It combines the concept of Scan by controlling the switching of radar antenna beams among subswaths to achieve wider coverage. Moreover, similar to Spotlight/Sliding Spotlight, Mosaic uses azimuth beam sweeping to prolong the target irradiation time and increase the resolution [16]. To satisfy the requirements of different tasks, more and more advanced spaceborne SARs have adopted the above modes, such as TerraSAR [21], Sentinel [22], and Gaofen-3 [23].

Nowadays, several matching filtering (MF)-based methods have been proposed for processing data from different SAR imaging modes [24,25,26]. In 1996, Moreira et al. introduced an extended chirp scaling (ECS) method, which employs a spectral analysis technique to process ScanSAR data [27]. Building on ECS, Prats et al. developed the ECS–baseband azimuth scaling (BAS) algorithm in 2010, which utilizes the subaperture technique to address the issue of Doppler spectrum aliasing [28]. While these methods process raw data computationally efficiently, they still face challenges in data acquisition, storage, and transmission, especially for high-resolution wide-swath imaging. In addition, they cannot be used to process echoes that do not satisfy the Shannon–Nyquist sampling theorem [29,30]. The developed sparse SAR imaging technology offers a solution to deal with the above problems. Compared with MF-based methods, sparse SAR imaging algorithms can achieve high-quality imaging with lower pulse repetition frequency (PRF) and obtain images with higher performance. The concept of sparse signal processing was introduced into SAR imaging in 2001 [31]. In 2006, Donoho et al. proposed compressive sensing (CS) [32], which challenges the traditional Shannon–Nyquist sampling theory [33] and enables the reconstruction of sparse signals from a reduced number of samples. In 2012, Zhang et al. proposed an approximated observation-based sparse SAR imaging method, which has lower computational complexity than conventional observation matrix-based algorithms, allowing large-scale sparse scene recovery [34]. In the following years, this technology has been further developed and applied to various SAR imaging modes, including Stripmap [35], Scan [36,37], Sliding Spotlight [38], and TOPS [39,40]. Owing to its advantage of wider swath width, sparse SAR imaging shows another option for high-resolution and wide-swath imaging.

Motivated by the performance advantages of sparse SAR, this paper proposes a mode design scheme for spaceborne sparse SAR imaging. In the proposed scheme, we first design the beam positions of sparse imaging mode based on its corresponding traditional mode. Then, the basic system parameters of the designed sparse mode are calculated based on the radar equations. Subsequently, the AAP and system performance are analyzed to verify the superiority of the novel mode. Finally, the mixed-norm regularization-based sparse SAR imaging method is introduced to obtain a high-quality image with less azimuth ambiguity from the data collected by the designed sparse mode. The experimental results and performance analysis show that compared with the traditional mode, the corresponding sparse mode obtains a wider swath coverage by reducing PRF, without changing the existing on-board system’s hardware conditions. In addition, it can suppress the effect of antenna beam scanning on system performance and has lower range ambiguity. Different from MF-based methods, the sparse imaging algorithm can obtain images with higher quality when the echo data used is downsampled, and it can achieve unambiguous recovery of large-scale surveillance areas with a higher image signal-to-clutter-noise ratio (SCNR).

This paper is organized as follows: Section 2 introduces the traditional SAR imaging mode briefly, such as the observation geometry and signal characteristics. In Section 3, we demonstrate the design principle of the sparse imaging mode in detail, including beam position design, parameter calculation, AAP, system performance analysis, and the mixed-norm regularization-based sparse SAR imaging method. Experimental results are shown in Section 4 to verify the designed framework. Finally, conclusions are drawn in Section 5.

## 2. Traditional SAR Imaging Mode

### 2.1. Observation Geometry

**Figure 1:**
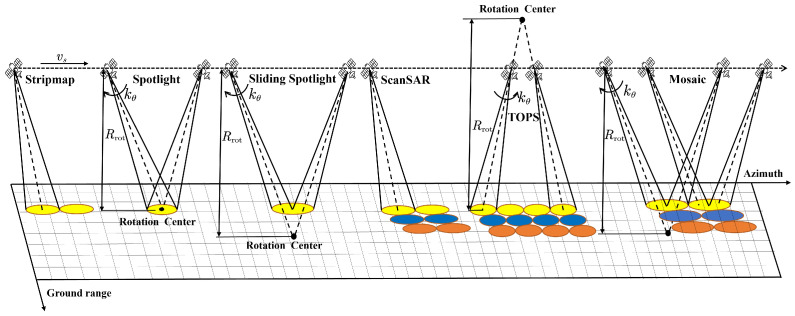
The acquisition geometry of all kinds of typical SAR imaging modes.

**Figure 2:**
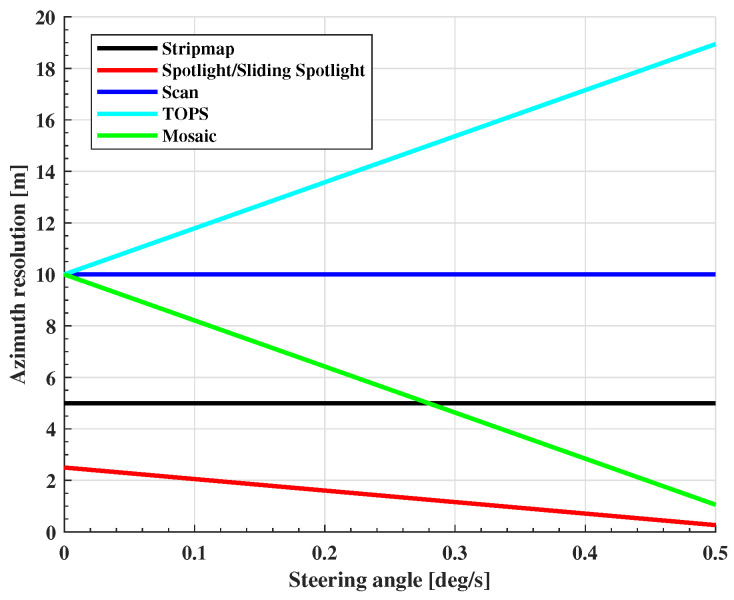
Azimuth resolution of each imaging mode.

The imaging geometry of all kinds of SAR imaging modes is depicted in Figure 1. To further discuss the difference between all kinds of modes, we introduce a mixing degree factor:

(1) $$\begin{array}{c} A\;=\;\frac{R_{\mathrm{rot}}-R_{0}}{R_{\mathrm{rot}}}\;=\;1-\frac{R_{0}k_{\theta }}{v_{g}} \end{array}$$

where $R_{\mathrm{rot}}$ is the distance from SAR platform to the rotation center, $R_{0}$ is the slant range, $k_{\theta }$ is the steering angle rate, and $v_{g}$ is the ground velocity. When A=0, it indicates that the platform is working in Spotlight mode. If A=1, this means that $R_{\mathrm{rot}}=\infty$, indicating that SAR is working in Stripmap or Scan mode. In TOPS, $k_{\theta }$ is negative and A>1, while in Sliding Spotlight and Mosaic mode, $k_{\theta }$ is positive and 0<A<1. Based on the mixing degree factor, we can obtain the theoretical resolution of all kinds of modes, i.e.,

(2) $$\begin{array}{c} \rho \;\approx \;\frac{L_{a}}{2}\left(N_{B}+1\right)A \end{array}$$

where $L_{a}$ is the azimuth antenna size, and $N_{B}$ is the number of subswaths. The relationship between the resolution of each mode and the steering angle rate is shown in Figure 2 (assuming that the number of subswaths is three). It is seen that with the steering angle rate increasing, Sliding Spotlight has the highest resolution, followed by Mosaic, Stripmap, and Scan, with TOPS performing the worst, and the resolution of Mosaic can be higher than that of Stripmap.

### 2.2. Signal Characteristics

The echo data y(t,τ) of different SAR imaging modes at time (t,τ) can be written as

(3) $$\begin{array}{cc} y(t,\tau )\; & =\;\underset{\left(p,q\right)}{\;\int \int \;}\sigma \left(p,q\right)\;\omega_{a}^{2}\left(\theta_{a}\right)\;\omega_{r}^{2}\left(\theta_{r}\right)\;\mathrm{rect}\left(\frac{\tau -2R/c}{T_{p}}\right)\\ \; & \;\cdot \;\exp\left\{-j\frac{4\pi }{\lambda }R\right\}\cdot \exp\left\{-j\pi K_{r}{\left(\tau -\frac{2R}{c}\right)}^{2}\right\}dpdq \end{array}$$

where *t* and τ are the azimuth and range time, *p* and *q* are the target azimuth and range positions, respectively, σ(p,q) is the backscattering coefficient, *c* is the speed of light, $\omega_{a}\left(\theta_{a}\right)$ and $\omega_{r}\left(\theta_{r}\right)$ are the azimuth and range antenna pattern weighting, $\theta_{a}$ and $\theta_{r}$ are the azimuth angle and range angle between the direction to the target and the antenna’s electrical axis, respectively, $T_{p}$ is the pulse duration, λ is the wavelength, $K_{r}$ is the linear frequency modulated rate, $R=\sqrt{r_{0}^{2}+{\left(v_{r}t-x\right)}^{2}}$ is the slant range of the target located at $\left(x,r_{0}\right)$ with the azimuth position *x* and the closest slant range $r_{0}$, and $v_{r}$ is the effective radar velocity. It should be noted that due to the different modes of beam scanning, the azimuth antenna pattern is also expressed differently. The Doppler center frequency of each mode can be expressed as

(4) $$\begin{array}{c} f_{ac}\left(t\right)\;=\;\frac{2v_{r}}{\lambda }\sin\left(\theta_{ac}\left(t\right)\right) \end{array}$$

where $\theta_{ac}\left(t\right)$ is the effective squint angle between the direction to the target and the antenna’s electrical axis. In Stripmap and Scan modes, considering a subswath, $\theta_{ac}\left(t\right)$ is a fixed value because the beam is not scanned in the azimuth direction. In Spotlight, Slide Spotlight, and TOPS modes, the Doppler center frequency varies with azimuth time when scanning the beam along the azimuth direction. The change rate of the Doppler center frequency can be written as

(5) $$\begin{array}{c} K_{c}\;=\;\frac{df_{ac}\left(t\right)}{dt}\approx \frac{2v_{s}}{\lambda }k_{\theta } \end{array}$$

where $v_{s}$ is the SAR velocity. It is seen that the Doppler center varies linearly when the beam is scanned at a constant speed.

In the following, we conduct an analysis of the azimuth ambiguity-to-signal ratio (AASR) and range ambiguity-to-signal ratio (RASR) for all imaging modes. The occurrence of AASR arises from the finite PRF and the periodic repetition of the Doppler spectrum of the discrete signal after sampling. In Stripmap, the AASR is

(6) $$\begin{array}{c} {\mathrm{AASR}}_{\mathrm{St}}\;=\;\frac{\sum_{m=-\infty }^{m=+\infty }\int_{-B_{D}/2}^{B_{D}/2}G{\left(f_{a}+m\cdot \mathrm{PRF}\right)}^{2}df_{a}}{\int_{-B_{D}/2}^{B_{D}/2}G{\left(f_{a}\right)}^{2}df_{a}} \end{array}$$

where $B_{D}$ is the Doppler bandwidth, G(·) is the AAP, and $f_{a}$ is the azimuth frequency. In Stripmap, it is seen that the AASR is associated with parameters such as PRF and Doppler bandwidth, and it is independent of the azimuth position.

For Spotlight, the AASR is expressed as

(7) $$\begin{array}{c} {\mathrm{AASR}}_{\mathrm{Sp}}\;=\;\frac{\sum_{m=-\infty }^{m=+\infty }\int_{-B_{D}/2-K_{a}t_{0}}^{B_{D}/2-K_{a}t_{0}}G{\left(m\cdot \mathrm{PRF}-K_{a}t_{0}\right)}^{2}df_{a}}{\int_{-B_{D}/2-K_{a}t_{0}}^{B_{D}/2-K_{a}t_{0}}G{\left(-K_{a}t_{0}\right)}^{2}df_{a}} \end{array}$$

where $t_{0}$ is the zero-Doppler moment of the target, and $K_{a}$ is the Doppler-modulated frequency. It can be observed that in Spotlight mode, the AASR is dependent on the azimuth position of the target. For Scan mode, the varying weights in the AAP received at different azimuth positions contribute to distinct AASR. Therefore, the AASR is

(8) $$\begin{array}{c} {\mathrm{AASR}}_{\mathrm{Sc}}\;=\;\frac{\sum_{m=-\infty }^{m=+\infty }\int_{-B_{D}/2-K_{a}t_{0}}^{B_{D}/2-K_{a}t_{0}}G{\left(f_{a}+m\cdot \mathrm{PRF}\right)}^{2}df_{a}}{\int_{-B_{D}/2-K_{a}t_{0}}^{B_{D}/2-K_{a}t_{0}}G{\left(f_{a}\right)}^{2}df_{a}}. \end{array}$$

For TOPS, Sliding Spotlight, and Mosaic modes, the AASR can be written as

(9) $$\begin{array}{c} {\mathrm{AASR}}_{T,S,M}\;=\;\frac{\sum_{m=-\infty }^{m=+\infty }\int_{-B_{D}/2+f_{ac}}^{B_{D}/2+f_{ac}}G{\left({f_{a}}^{\prime }+m\cdot \mathrm{PRF}\right)}^{2}df_{a}}{\int_{-B_{D}/2+f_{ac}}^{B_{D}/2+f_{ac}}G{\left({f_{a}}^{\prime }\right)}^{2}df_{a}} \end{array}$$

with

(10) $$\begin{array}{c} {f_{a}}^{\prime }\;=\;\left(1-\frac{K_{c}}{K_{a}}\right)\left(f_{a}-f_{ac}\right). \end{array}$$

The expressions of AASR are identical for these three modes. The difference lies in the sign of the steering angle rate. TOPS has a positive rate. Sliding Spotlight and Mosaic have a negative one. In summary, it can be concluded that the differences in AASR among various modes arise from variations in the AAP, which result from different antenna scanning mechanisms employed in each mode.

The causes of RASR in each imaging mode are similar, and its expression can be given by

(11) $$\begin{array}{c} \mathrm{RASR}\;=\;\frac{\sum_{m\neq 0}\frac{{G_{r}}^{2}\left(\theta_{r}\left(r+m\cdot c/\mathrm{PRF}/2\right),\eta \right)\cos\left[\theta_{i}\left(r+m\cdot c/\mathrm{PRF}/2\right)\right]}{{\left(r+m\cdot c/\mathrm{PRF}/2\right)}^{3}\sin\left[\theta_{i}\left(r+m\cdot c/\mathrm{PRF}/2\right)\right]}}{\frac{{G_{r}}^{2}\left(\theta_{r},\eta \right)\cos\left[\theta_{i}\left(r\right)\right]}{r^{3}\sin\left[\theta_{i}\left(r\right)\right]}} \end{array}$$

where $\theta_{i}\left(r\right)$ is the incidence angle of *r*, and $G_{r}\left(\cdot \right)$ is the range AAP. It is noted that in imaging modes with multiple subswaths, i.e., Scan, TOPS, and Mosaic, each subswath has a different PRF and incidence angle. Therefore, the RASR is different for each subswath.

## 3. Design and Performance Analysis of Sparse SAR Imaging Mode

In this section, we will introduce the design scheme of the sparse SAR imaging mode including beam position design, parameter calculation, and system performance analysis in detail. Sparse SAR can obtain high-quality images from downsampled data. Thus, the sparse imaging mode has a lower requirement of PRF than the corresponding conventional one, and it can obtain wider swath coverage.

### 3.1. Beam Position Design

**Figure 3:**
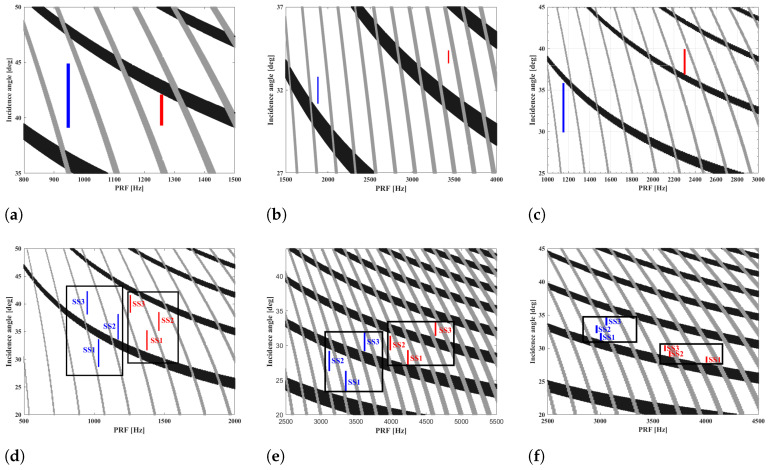
Timing diagram of the designed sparse SAR imaging modes. (**a**) Sparse Stripmap. (**b**) Sparse Spotlight. (**c**) Sparse Sliding Spotlight. (**d**) Sparse Scan with three subswaths. (**e**) Sparse TOPS with three subswaths. (**f**) Sparse Mosaic with three subswaths.

The selection of beam position plays a critical role in the system design. The choice of PRF takes into account the limitations of transmit and nadir interference. The timing diagrams depicted in Figure 3 present the beam position designs for Stripmap, Spotlight, Sliding Spotlight, Scan, TOPS, and Mosaic, where the blue line represents the sparse imaging mode, and the red one represents the corresponding traditional one. The beam position design of these sparse modes is based on PRFs that are 75%, 50%, 50%, 75%, 75%, and 75% of their corresponding conventional ones, respectively. The selection of the downsampling ratio for sparse imaging modes depends on the practical application. In the following, we take Sliding Spotlight (high-resolution mode) and Scan (wide-swath mode) as examples to analyze the designed system’s performance.

### 3.2. System Performance Analysis of Sliding Spotlight Mode

Firstly, the system performances of both traditional and designed sparse modes are calculated. Then, the relationship between AASR and PRF at different azimuth steering angles is analyzed. This section analyzes the effect of azimuth steering angle on AASR based on the system parameters of both conventional and sparse Sliding Spotlight modes. Finally, we analyzed the change in RASR with PRF and compared the RASR values using the system parameters of these two modes.

**System Parameter Calculation**. According to Figure 3c, the basic system parameters of the sparse and traditional Sliding Spotlight modes are calculated in Table 1. The swath width of the traditional mode is 45.03 km, and PRF=2300Hz, while in the design of sparse Sliding Spotlight, PRF is reduced to 1150 Hz to obtain the 90.16 km swath width.

**Grating Lobes Analysis**. In order to achieve antenna beam sweeping, the Sliding Spotlight mode usually adopts the phased array antenna. The AAP of total antenna is

(12) $$\begin{array}{c} G\left(\theta ,t\right)\;=\;G_{e}\left(\theta \right)\cdot G_{T}\left(\theta ,t\right)\cdot G_{R}\left(\theta ,t\right) \end{array}$$

where θ is the azimuth illumination angle, and $G_{e}\left(\theta \right)$ is the single-element AAP, which is written as

(13) $$\begin{array}{c} G_{e}\left(\theta \right)\;=\;{\mathrm{sinc}}^{2}\left(\frac{L_{e}}{\lambda }\sin\theta \right). \end{array}$$

**Figure 4:**
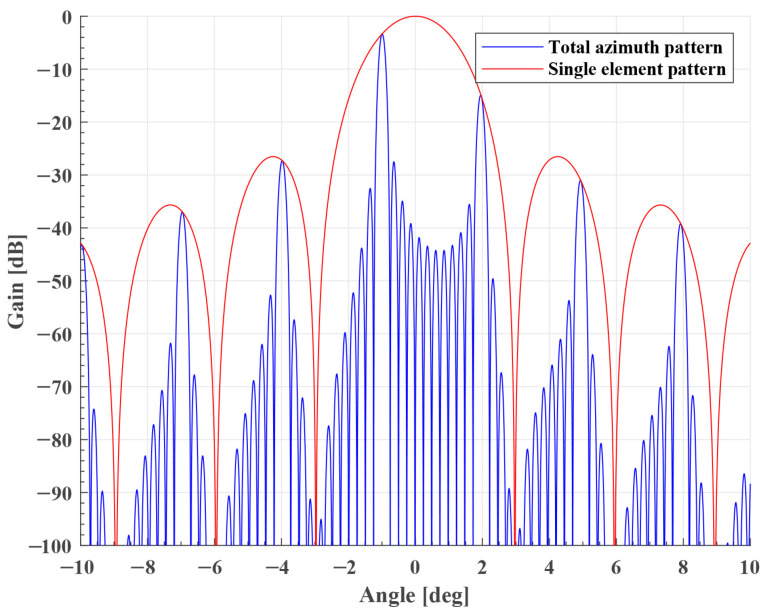
Element and total AAP of Sliding Spotlight mode. Steering angle is equal to 1°.

$G_{T}\left(\theta ,t\right)$ and $G_{R}\left(\theta ,t\right)$ are the transmit and receive AAP, i.e.,

(14a) $$\begin{array}{cc} G_{T}\left(\theta ,t\right)\; & =\;\left|\frac{1}{M}\sum_{k=0}^{M-1}C_{T}\exp\left(j\frac{2\pi k}{\lambda }L_{e}\sin\theta \right)\right| \end{array}$$

(14b) $$\begin{array}{cc} G_{R}\left(\theta ,t\right)\; & =\;\left|\frac{1}{M}\sum_{k=0}^{M-1}C_{R}\exp\left(j\frac{2\pi k}{\lambda }L_{e}\sin\theta \right)\right| \end{array}$$

where *M* is the number of transmit and receive modules, $L_{e}=L_{a}/M$ is the single-element antenna size, $L_{a}$ is the total antenna size, $C_{T}$ and $C_{R}$ are the transmit and receive excitation coefficients, i.e.,

(15a) $$\begin{array}{cc} C_{T} & =a_{T}\cdot \exp\left(j\frac{2\pi k}{\lambda }L_{e}\sin\phi \left(t\right)\right) \end{array}$$

(15b) $$\begin{array}{cc} C_{R} & =a_{R}\cdot \exp\left(j\frac{2\pi k}{\lambda }L_{e}\sin\phi \left(t+\Delta t\right)\right) \end{array}$$

where Δt is the delay time, and $a_{T}$ and $a_{R}$ are the amplitude of transmit and receive excitation coefficients, respectively. We can analyze the grating lobes of the Sliding Spotlight mode by two-way AAP. Based on the equations above, it is found that the AAP is affected by the steering angle in Sliding Spotlight. The total and single-element AAPs are shown in Figure 4. The sidelobes are proportional to the steering angle, which means that the azimuth beam scanning will affect the system’s performance. In practice, we expect the system’s performance to be unaffected by azimuth beam scanning.

**Figure 5:**
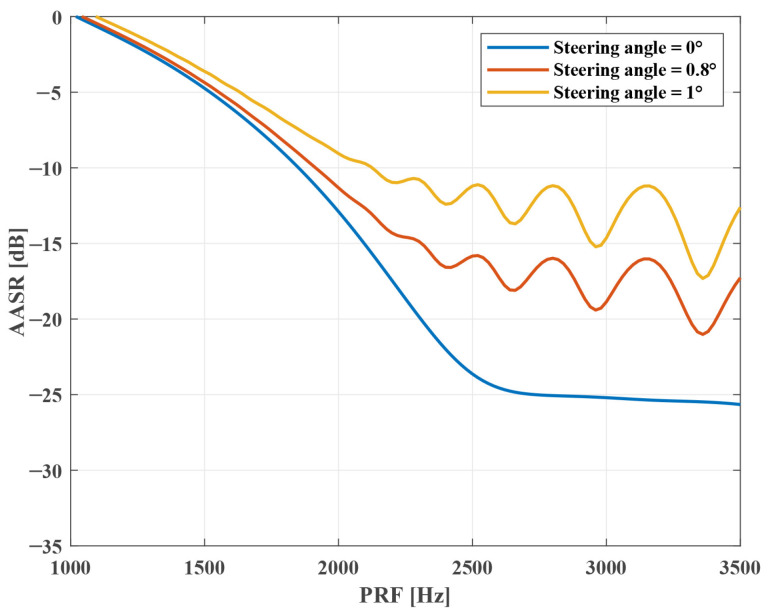
AASR versus PRF under different steering angles in Sliding Spotlight.

**Figure 6:**
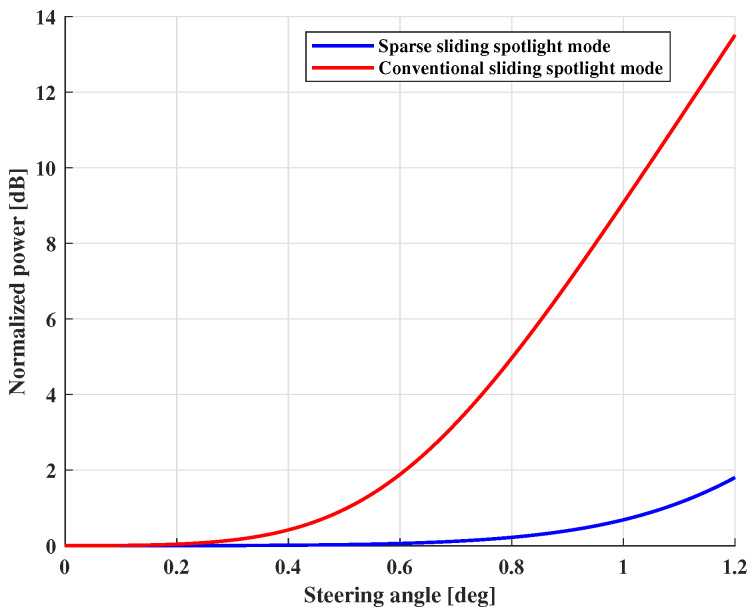
AASR deterioration versus steering angle in Sliding Spotlight. The power loss is normalized to the boresight case.

**Azimuth Ambiguity Analysis**. Figure 5 shows the AASR versus PRF under different steering angles. The results demonstrate that AASR is inversely related to PRF, and the steering angle has an effect on AASR. Moreover, the change in AASR becomes more obvious with the PRF increasing. In addition, it is found that AASR increases by about 15.2 dB in sparse Sliding Spotlight mode, resulting in serious azimuth ambiguity. But the mixed-norm regularization-based sparse SAR imaging method can solve this problem by introducing the azimuth ambiguity terms into the model, which will be presented in Section 3.4. Figure 6 illustrates the change in AASR with the steering angle. The power loss is normalized to the boresight case. It is seen that when the steering angle is above 1°, AASR deteriorates by about 8.6 dB in traditional Sliding Spotlight mode. But if steering angle =1.2°, the change in AASR is less than 2 dB in sparse mode. Thus, the sparse Sliding Spotlight mode effectively reduces the effect on AASR from a steering angle (antenna beam azimuth scanning), which means that it can achieve higher-quality images compared to the traditional mode.

**Figure 7:**
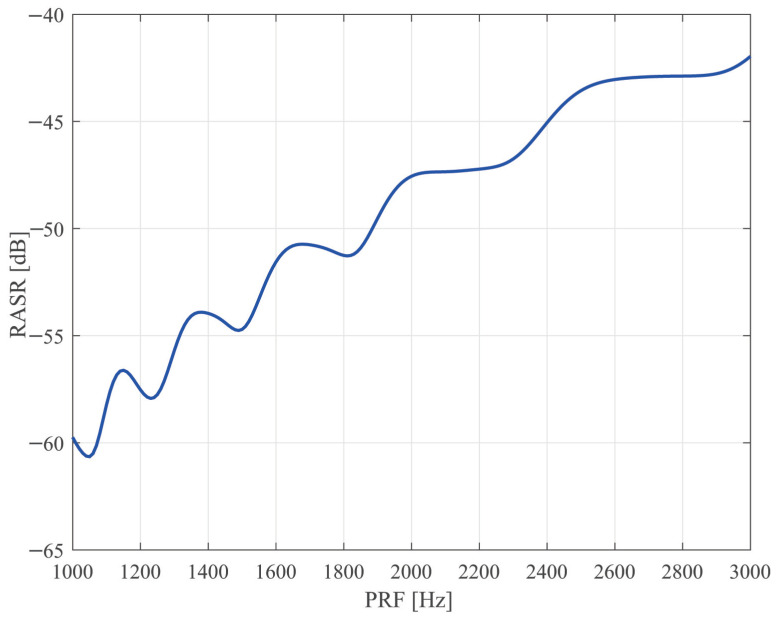
The relationship between RASR and PRF in Sliding Spotlight.

**Range Ambiguity Analysis**. Figure 7 shows the variation in RASR with PRF. From Figure 7, it is seen that the sparse Sliding Spotlight mode can reduce RASR by about 10.7 dB. This indicates that it can obtain a high-quality SAR image.

In summary, compared with the traditional Sliding Spotlight mode, the designed sparse mode has a wider swath coverage at lower PRF. The system performance analysis results indicate that the sparse Sliding Spotlight mode can mitigate the deterioration of AASR with increasing steering angle and significantly reduce RASR.

### 3.3. System Performance Analysis of Scan Mode

**System Parameter Calculation**. According to the timing diagram in Figure 3d, we compare the essential system parameters of sparse and traditional Scan modes. The basic system parameters of sparse and traditional Scan modes are calculated in Table 2. To illustrate the differences between them, we focus on subswath 1 (SS1) as the example. In the traditional mode, SS1 has a swath width of 60.02 km with a PRF of 1375 Hz. In contrast, the designed sparse Scan mode reduces PRF to 1031 Hz, resulting in a wider swath width of 80.05 km. By reducing the PRF to 75% of its original value, the swath coverage of each subswath in sparse Scan mode increases from 60 km to 80 km. This leads to a total swath width increase from 180 km to 240 km, without any changes to other parameters such as azimuth resolution and beamwidth.

**Figure 8:**
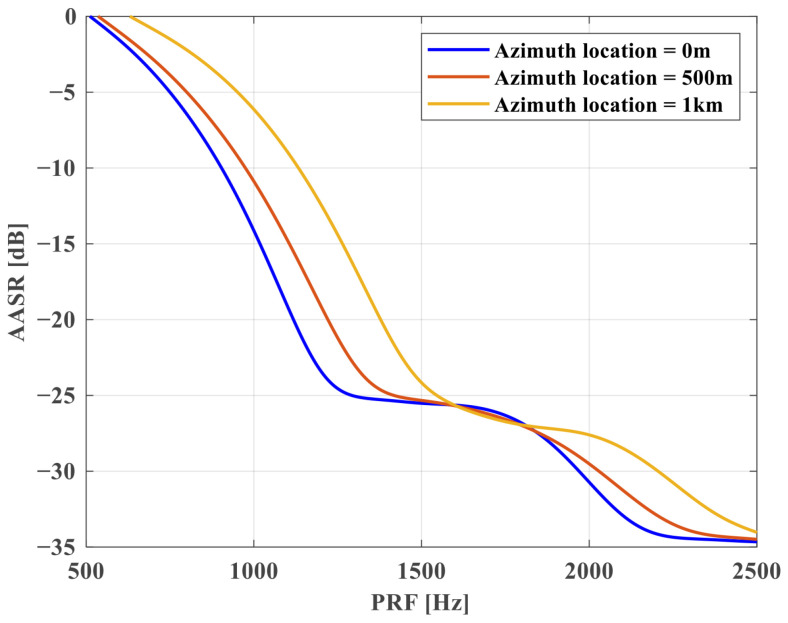
AASR versus PRF at different azimuth positions in Scan mode.

**Azimuth Ambiguity Analysis**. The relationship between AASR and PRF for SS1 in Table 2 is shown in Figure 8, and the corresponding variation curves are given for azimuth positions at 0 m, 500 m, and 1 km, respectively. From Figure 8, it can be seen that the AASR value increases with the azimuth position. In addition, because of the reduced PRF, AASR deteriorates from −16∼−25 dB to −6∼−14 dB. Thus, it is necessary to suppress the azimuth ambiguity using the sparse imaging method.

**Figure 9:**
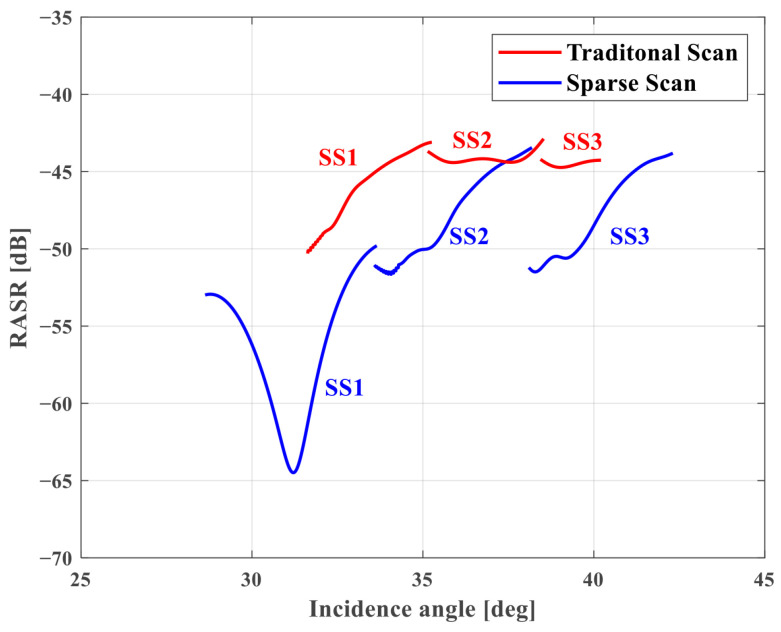
The relationship between RASR and incidence angle in Scan mode.

**Figure 10:**
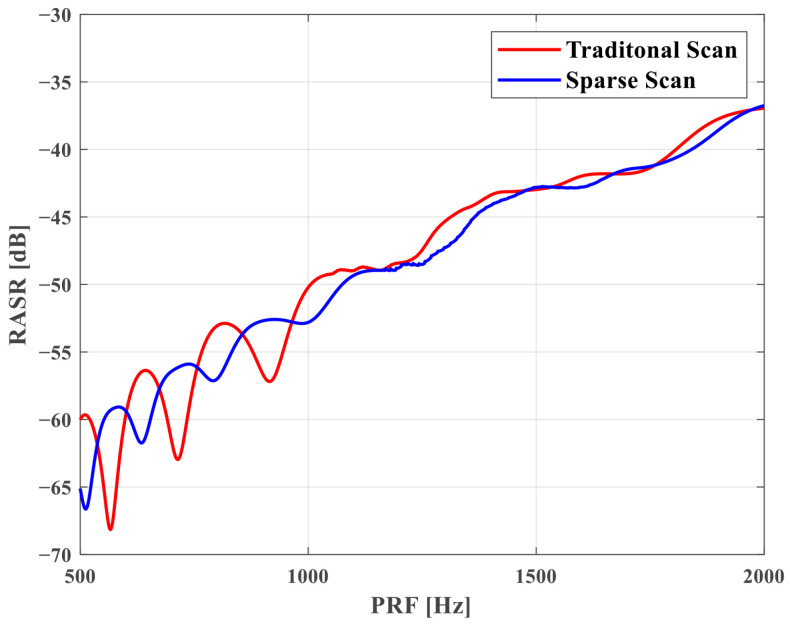
The relationship between RASR and PRF in Scan mode.

**Range Ambiguity Analysis**. The RASR of the Scan mode is calculated in the same way as in Stripmap, as the Scan mode has no extra processing in the range direction. However, since the PRF is different for each subswath, the RASR of each subband is also different. The relationship between RASR and the incidence angle for each subswath in traditional and sparse Scan modes is shown in Figure 9. The red line is the traditional Scan mode, and the blue line is the sparse one. It is seen that the RASR of each subswath of the sparse mode is lower than that of the traditional one. Then, we take SS1 for example to analyze RASR. The relationship between RASR and PRF for SS1 in Table 2 is shown in Figure 10. It is seen that in the sparse mode, the RASR varies from −43.9 dB to −51.9 dB due to changes in the incidence angle and PRF, which indicates that the range ambiguity is suppressed in the sparse mode.

### 3.4. Mixed-Norm Regularization-Based Sparse SAR Imaging

$L_{q}$**-norm Regularization-Based Sparse Imaging.** Let $N_{P}$ and $N_{Q}$ denote the number of points in the azimuth and range directions, and let $X\in C^{N_{P}\times N_{Q}}$ and $Y\in C^{N_{t}\times N_{\tau }}$ denote the backscattered coefficient of the surveillance region and echo data, respectively. $I\left(\cdot \right)$ and $G\left(\cdot \right)$ are the imaging and inverse imaging operators of the MF-based algorithm. The echo data under sparse parameters can be regarded as the echo data received under traditional parameters with azimuth downsampling. Then, the approximated observation-based sparse SAR imaging model can be expressed as

(16) $$\begin{array}{c} Y\;=\;H\circ G(X)\;+\;N \end{array}$$

where H is the downsampling matrix in the azimuth direction, N is the noise, and ∘ is the Hadamard product operator. For the model in (16), the considered scene can be reconstructed by solving the $L_{q}\left(0<q\leq 1\right)$-norm regularization problem [34,39], i.e.,

(17) $$\begin{array}{c} \hat{X}\;=\;\arg\min_{X}\left\{{∥Y-H\circ G(X)∥}_{F}^{2}\;+\;\beta {∥X∥}_{q}\right\} \end{array}$$

where $\hat{X}$ is the reconstructed image of the considered 2-D scene, and β is the regularization parameter.

$L_{2,q}$**-norm Regularization-Based Sparse Imaging.** The above $L_{q}$-norm regularization-based sparse imaging method can recover the large-scale surveillance area, but it fails to achieve ambiguity suppression. The sparse SAR imaging mode inevitably produces azimuth ambiguity due to the adoption of a downsampled beam position. To achieve high-quality sparse imaging in the designed modes, an $L_{2,q}$-norm regularization-based method is proposed and introduced for the data processing of the collected echo data. Because the azimuth ambiguities are considered in the construction of the imaging model, the proposed method can achieve ambiguity suppression and hence obtain the unambiguous sparse image of the considered scene. The unambiguous imaging model can be written as [41]

(18) $$\begin{array}{c} Y\;=\;H\circ \left(G\left(X\right)+\sum_{i}G_{i}\left(X_{i}\right)\right)\;+\;N,\;i\in Z,i\neq 0 \end{array}$$

where $G_{i}\left(\cdot \right)$ refers to the inverse imaging operators for ambiguity areas, and $X_{i}\in C^{N_{P}\times N_{Q}}$ with $i\in Z^{-}$ and $i\in Z^{+}$ are the left and right ambiguity areas. Then, the considered scene can be recovered by solving the $L_{2,q}$-norm regularization problem, i.e.,

(19) $$\begin{array}{c} \hat{X}\;=\;\arg\min_{X}\left\{{∥Y-H\circ \left(G\left(X\right)+\sum_{i}G_{i}\left(X_{i}\right)\right)∥}_{F}^{2}\right.\;+\;\beta_{1}{∥X_{\mathrm{all}}∥}_{2,q}^{q}\;+\;\beta_{2}{∥X∥}_{1}\} \end{array}$$

where $\hat{X}$ is the reconstructed image of the considered scene, $\beta_{2}$ is the regularization parameter which controls the sparsity of X, and $\beta_{1}$ controls the sparsity of the whole scene $X_{\mathrm{all}}$, which can be expressed as

(20) $$\begin{array}{c} {∥X_{\mathrm{all}}∥}_{2,q}^{q}={\left(\sum_{np=1}^{N_{p}}{\left(\left|X_{np}\right|+\sum_{i}\left|X_{i,np}\right|\right)}^{q}\right)}^{1/q} \end{array}$$

where $X_{np}$ and $X_{i,np}$ are the npth rows of X and $X_{i}$, and $np=1,2,\cdots ,N_{P}$. The detailed iterative process of solving the regularization problem in (20) is depicted in [41] when q=1. Then, the focused unambiguous image can be obtained. The $L_{2,q}$-norm regularization-based method has also been applied to other sparse modes, such as TOPS [40]. The convergence analysis of this method is detailed in [42].

## 4. Experiments and Discussion

**Figure 11:**
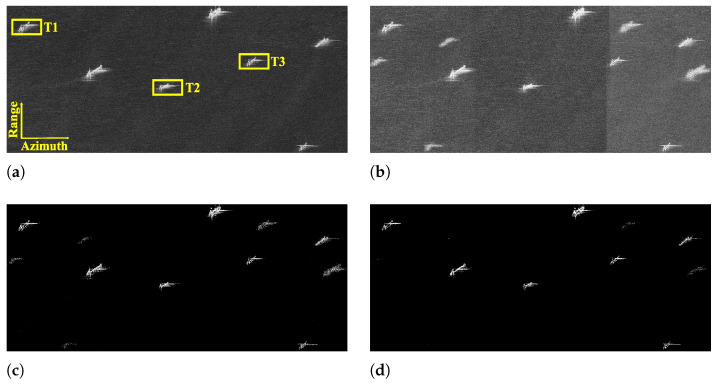
Reconstructed images of ships by different methods from the data collected by the sparse Sliding Spotlight mode. (**a**) MF (traditional parameters). (**b**) MF. (**c**) $L_{q}$-norm regularization-based sparse imaging method. (**d**) $L_{2,q}$-norm regularization-based sparse imaging method.

**Figure 12:**
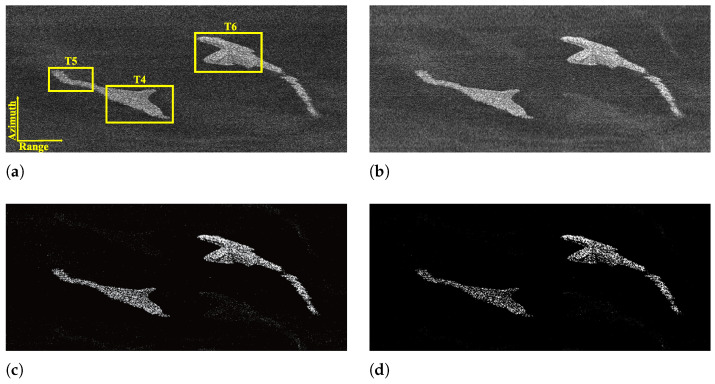
Reconstructed images of islands by different methods from the data collected by the sparse Scan mode. (**a**) MF (traditional parameters). (**b**) MF. (**c**) $L_{q}$-norm regularization-based sparse imaging method. (**d**) $L_{2,q}$-norm regularization-based sparse imaging method.

To validate the performance of the designed modes, some experiments are performed based on the parameters of the designed sparse Sliding Spotlight and Scan modes (see Table 1 and Table 2). The experiments are implemented in MATLAB version R2023b and on a computer with a 3.0 GHz Intel i9 processor and an NVIDIA GeForce RTX 4090 GPU. In the experiment of the Sliding Spotlight imaging mode, the computation times of MF, the $L_{q}$-norm regularization-based method, and the $L_{2,q}$-norm regularization-based method are 11.62 s, 74.67 s, and 230.92 s, respectively. For the Scan mode experiment, the running times of above three methods are 1.62 s, 10.13 s, and 27.68 s, respectively, which are consistent with the theoretical analysis of computational complexity in [40]. In the experiments, selecting an appropriate iteration step size enabled satisfactory results to be achieved within five iterations. Figure 11 and Figure 12 show the recovered images of different kinds of surveillance areas by MF, $L_{q}$-norm regularization-based and $L_{2,q}$-norm regularization-based methods from the collected echo data of the designed sparse modes (q=1). For comparison, Figure 11a and Figure 12a depict the MF-based images recovered from the data collected by the traditional Sliding Spotlight and Scan modes (parameters are in Table 1 and Table 2). In Scan mode, the parameters of the SS1 subswath are used as an example for verification. Since the PRF satisfies the requirements of the sampling theorem, it is found that MF can effectively achieve high-quality recovery of the considered sparse scenes. But for the designed sparse modes, it fails to recover the sparse scenes with obvious ambiguities and energy dispersion along the azimuth direction (see Figure 11b and Figure 12b). Compared with MF-based results, as shown in Figure 11c and Figure 12c, the recovered images of the $L_{q}$-norm regularization-based method are well focused with better performance. However, they still suffer from the azimuth ambiguities, which lead to false alarms and inaccurate interpretation. In comparison, the $L_{2,q}$-norm regularization-based sparse imaging method consistently achieves unambiguous recovery of the surveillance region, even when the data are collected with a reduced PRF in sparse modes (see Figure 11d and Figure 12d). To further verify the proposed method, the target-to-ambiguity ratio (TAR) and target-to-background ratio (TBR) are introduced to quantitatively evaluate the ambiguity and noise suppression ability of different methods, which are defined as

(21) $$\begin{array}{c} \mathrm{TAR}\;≜\;10\log_{10}\left(\frac{\sum_{(np,nq)\in M}{\left|X(np,nq)\right|}^{2}}{\sum_{\left(np,nq\right)\in A_{i}}{\left|X(np,nq)\right|}^{2}}\right) \end{array}$$

(22) $$\begin{array}{c} \mathrm{TBR}\;≜\;10\log_{10}\left(\frac{\sum_{(np,nq)\in T}{\left|X(np,nq)\right|}^{2}}{\sum_{(np,nq)\in B}{\left|X(np,nq)\right|}^{2}}\right) \end{array}$$

where M denotes the main imaging area, $A_{i}$ is the *i*th azimuth ambiguity area with $i\in Z^{-}$ and $i\in Z^{+}$ being the shifting indexes of ambiguity areas in the left and right sides of the main imaging area in the azimuth direction, respectively, and B indicates the background region around the target area T. The TAR and TBR values of the recovered images in Figure 11 and Figure 12 are shown in Table 3, respectively. From Table 3, it is seen that compared with MF and the $L_{q}$-norm regularization-based methods, the $L_{2,q}$-norm regularization-based sparse SAR imaging method suppresses the azimuth ambiguity dramatically in the designed sparse modes and hence obtains images with higher SCNR.

## 5. Conclusions

In this paper, the design scheme of a sparse SAR imaging mode is described in detail. It combines the advantages of traditional imaging modes and sparse signal processing. Compared with traditional modes, the designed sparse modes utilize downsampled beam positions and adopt a sparse imaging method, achieving wider swath coverage, reducing data volume, and lowering RASR without hardware changes. The used $L_{2,q}$-norm regularization-based sparse SAR imaging method can obtain unambiguous images of large-scale areas with reduced PRF, which makes it possible to achieve high-quality data processing in spaceborne sparse SAR systems. Sparse SAR imaging performance relies on prior scene knowledge, generally assuming sparsity. Thus, it should be noted that the proposed design scheme of sparse SAR imaging modes is more applicable to the observation of sparse surveillance areas.

## Figures and Tables

**Table 1:** The parameters of designed sparse and traditional Sliding Spotlight modes.

	Sparse	Traditional
PRF [Hz]	1150	2300
Pulse duration [ms]	0.02	0.02
Incident angle (near) [deg]	32.32	36.87
Incident angle (far) [deg]	38.15	39.94
Middle look angle [deg]	35.24	38.41
Azimuth resolution [m]	0.5	0.5
Azimuth beamwidth [deg]	0.22	0.22
Platform velocity [m/s]	7600	7600
Maximum steering angle [deg]	±3.5	±3.5
Azimuth swath width [km]	45	45
Range swath width [km]	90.16	45.03

**Table 2:** Parameters of designed sparse Scan mode and traditional Scan mode.

	SS1	SS2	SS3
Sparse Scan imaging mode			
PRF [Hz]	1031	1170	950
Incident angle (near) [deg]	28.63	33.58	38.10
Incident angle (far) [deg]	33.65	38.19	42.31
Middle look angle [deg]	27.33	31.34	34.93
Platform velocity [m/s]	7465	7465	7465
Range resolution [m]	8	8	8
Azimuth resolution [m]	15	15	15
Subswath width [km]	80.05	80.15	80.14
Total swath width [km]	∼240		
Traditional Scan imaging mode			
PRF [Hz]	1375	1460	1257
Incident angle (near) [deg]	31.63	35.14	38.44
Incident angle (far) [deg]	35.26	38.53	40.21
Platform velocity [m/s]	7465	7465	7465
Range resolution [m]	8	8	8
Azimuth resolution [m]	15	15	15
Subswath width [km]	60.02	60.04	60.08
Total swath width [km]	∼180		

**Table 3:** TAR and TBR values of the images recovered by different methods in the designed sparse modes [dB].

Sliding Spotlight	TAR		TBR of T2	
	T1	T3	Traditional	Sparse
MF	5.53	2.35	10.22	10.22
$L_{q}$-norm	17.97	9.77	22.69	26.85
$L_{2,q}$-norm	23.67	12.69	41.88	46.42
**Scan**	**TAR**		**TBR of T5**	
	**T4**	**T6**	**Traditional**	**Sparse**
MF	4.94	5.35	7.45	6.94
$L_{q}$-norm	10.88	16.90	20.57	23.07
$L_{2,q}$-norm	20.88	24.83	30.51	34.01

## Data Availability

Data is contained within the article.
